# Long-Term Outcomes of the 150 mm Drug-Coated Balloon Cohort from the IN.PACT Global Study

**DOI:** 10.1007/s00270-022-03214-y

**Published:** 2022-07-21

**Authors:** Marianne Brodmann, Wouter Lansink, Katharina Guetl, Antonio Micari, Jeremiah Menk, Thomas Zeller

**Affiliations:** 1 Department of Internal Medicine, Medical University, Graz, Austria; 2 Department of Thoracic and Vascular Surgery, Vascular Center ZOL, Genk, Belgium; 3 Interventional Cardiology, University of Messina Hospital, Messina, Italy; 4 Medtronic, Minneapolis, MN USA; 5 Angiology Department, Universitäts-Herzzentrum Freiburg–Bad Krozingen, Bad Krozingen, Germany

**Keywords:** Drug-coated balloon, Target lesion revascularization, Femoropopliteal, Peripheral artery disease, Long lesions

## Abstract

**Purpose:**

Data on the long-term safety and effectiveness of drug-coated balloons (DCBs) for the treatment of long femoropopliteal atherosclerotic lesions in the real-world setting are rare. This study reports 3 year and 5 year outcomes of the pre-specified 150 mm balloon sub-cohort of the IN.PACT Global Study.

**Methods:**

The IN.PACT Global Study was a prospective, multicentre, international, single-arm study evaluating the performance of the IN.PACT Admiral DCB in real-world patients with femoropopliteal atherosclerotic disease. This pre-specified 150 mm DCB cohort analysis comprised 107 participants (111 lesions) with all target lesions treated with at least one 150 mm DCB.

**Results:**

Mean lesion length was 20.3 ± 9.2 cm; 18.0% had in-stent restenosis, 58.6% were totally occluded, and 17.1% were severely calcified. Through 60 months, the Kaplan–Meier estimate of freedom from clinically driven target lesion revascularization (CD-TLR) was 72.7% [95% confidence interval (CI):62.4%–80.5%]. The safety composite endpoint (freedom from device/procedure-related death through 30 days; freedom from target limb major amputation and clinically driven target vessel revascularization through 5 years) was 70.5%. The cumulative incidence of major amputation was 1.0% and all-cause mortality was 18.4% through 60 months. Freedom from CD-TLR rates in the provisional stented and non-stented subgroups through 36 months were 64.0% [95% CI: 46.1%–77.3%] and 81.9% [95% CI: 69.7%–89.6%] (log-rank *p* = 0.074), respectively.

**Conclusions:**

The results demonstrate sustained long-term safety of the 150 mm IN.PACT Admiral DCB for long femoropopliteal atherosclerotic lesions in real-world patients. In particular, the results show that DCB angioplasty is an effective revascularization modality in long complex lesions. *ClinicalTrials.gov* identifier: NCT01609296.

**Level of Evidence.:**

Level 3, Cohort Study.

**Supplementary Information:**

The online version contains supplementary material available at 10.1007/s00270-022-03214-y.

## Introduction

Endovascular strategies have been considered as first-line therapy for revascularization in patients with peripheral artery disease (PAD) and femoropopliteal lesions up to 25 cm in length [1]. Among these, the paclitaxel drug-coated balloon (DCB) has emerged as a significant innovation that provides effective clinical outcomes without a permanent scaffold. For femoropopliteal arterial lesions, several randomized controlled trials (RCTs) have demonstrated the superior performance of DCBs compared to standard percutaneous transluminal angioplasty (PTA) [2–7]. While these promising outcomes recognized DCBs as a preferred choice over PTA, the lesions included in most RCTs are limited to shorter and less complex lesions, while longer lesions are often excluded. Although single-arm studies suggest the potential of DCBs for long lesions through 1 and 2 year follow-up [8–10], there are no long-term data available in real-world settings. Moreover, data on long balloons for long lesions are not available

The IN.PACT Global Study was a large prospective study designed to evaluate the performance of the paclitaxel-coated IN.PACT Admiral DCB (Medtronic, Dublin, Ireland) for the treatment of real-world patients with atherosclerotic disease of the superficial femoral artery (SFA) and/or the entire popliteal artery. A prospective analysis of a pre-specified cohort of participants with long lesions treated with the 150 mm IN.PACT Admiral DCB within the IN.PACT Global Study is presented in this paper. Herein, we report the 3 year and 5 year outcomes of the 150 mm cohort.

## Methods

### Study Design

The IN.PACT Global Study, a prospective, multicenter, international, single-arm clinical study, was designed to assess the safety and effectiveness of a paclitaxel-coated DCB for the treatment of real-world patients. In the full cohort, 1535 participants were enrolled across 64 sites in 26 countries from Europe, the Middle East, Asia, North Africa, Australia, Canada, and Latin America from 2012 to 2014. Outcomes from the full clinical cohort through 3 years have been previously reported [11–13]. The study included participants with intermittent claudication and/or rest pain [Rutherford categories (RC) 2–4] because of obstructive disease of the femoropopliteal artery. Lesions were located in the full native SFA and/or the full popliteal artery (P1–P3). Minimal selection criteria were applied to better represent the patient profile treated in the actual clinical practice in accordance with real-world evidence [14]. The present analysis included a pre-specified as-treated cohort, in which all target lesions were treated with at least one 150 mm DCB, thus assessing the treatment effect of long balloons for long complex lesions. Participants were followed for a total of 60 months. Participants had hospital visit evaluations through 36 months. At 48 and 60 months, participants had phone follow-up and the occurrence of reintervention, adverse events, and health status were assessed.

An independent Clinical Events Committee (CEC) managed by Syntactx, New York, NY, USA adjudicated all major adverse events (MAEs) including clinically driven target lesion revascularizations (CD-TLRs), clinically driven target vessel revascularizations (CD-TVRs) major target limb amputation, thrombosis at the target lesion site, and deaths through 60 months after the index procedures.

The institutional review board or ethics committee at each study site approved the study protocol. Informed consent was obtained from all patients prior to enrollment. The study was conducted in accordance with the Declaration of Helsinki, good clinical practice guidelines, and applicable laws as specified by all relevant governmental bodies. The trial was registered on the National Institutes of Health website (*ClinicalTrials.gov* identifier: NCT01609296).

### Outcome Measures

Assessments through 36 months included freedom from CD-TLR; the safety endpoint, a composite of freedom from device- and procedure-related mortality through 30 days and freedom from major target limb amputation and CD-TVR within 36 months after the index procedure; primary sustained clinical improvement, defined as a sustained upward shift of at least 1 RC compared to baseline without the need for repeated TLR or surgical revascularization in amputation-free surviving patients; secondary sustained clinical improvement, defined as a sustained upward shift of at least 1 RC compared with baseline, including the need for repeated TLR or surgical revascularization in amputation-free surviving patients; and the incidence of MAEs (all-cause mortality, CD-TVR, major target limb amputation, and thrombosis at the target lesion site), CD-TLR, any TVR, and any TLR. Assessments through 60 months included the safety composite endpoint, and the incidence of MAEs. CD-TLR was defined as any reintervention within the target lesion(s) because of symptoms or drop of ankle-brachial index (ABI) of ≥ 20% or > 0.15 when compared with post-index procedure baseline ABI; CD-TVR was defined as any reintervention within the target vessel due to symptoms or a reduction in ABI ≥ 20% or > 0.15 when compared with the post-index procedure baseline ABI; severe calcification was defined as a circumference ≥ 180° on both sides of the vessel at the same location and lengths greater than or equal to half of the total lesion length [15]. All repeat interventions on the target limbs, including TLR, TVR, and the clinically driven status, were reviewed and adjudicated by the CEC.

### Statistical Analysis

All baseline demographics and clinical characteristics were summarized on a participant basis and lesion characteristics were summarized on a lesion basis, unless otherwise specified. For baseline characteristics, continuous variables are described as mean ± standard deviation; dichotomous and categorical variables are described as counts and proportions. The outcome analysis was performed at a patient level. The Kaplan–Meier method was used to evaluate time-to-event data for freedom from CD-TLR and freedom from mortality through 60 months and cumulative incidence for other outcomes where applicable. The Kaplan–Meier analysis for CD-TLR by provisional stent usage was truncated at 1080 days because fewer than 30 patients were in the stent group at the start of 1080 days. Confidence intervals (95%) for the Kaplan–Meier method were derived using the log–log method. The difference in the survival curves between subgroups was assessed using the log-rank test. Time to event was also summarized using the restricted mean survival time (RMST) with a horizon of 1080 days and 1800 days for 3 years and 5 years, respectively. The RMST is the average time to an event within a fixed time period and corresponds to the area under the survival curve from the start of follow-up to the fixed time point. It incorporates participants with events, censoring, and those with complete follow-up through the time period without an event. Estimates are presented with the 95% confidence interval (CI) where applicable. All summaries were based on non-missing assessments. Time is presented in years and months where 1 year equals 360 days and one month equals 30 days. A significance level of 0.05 was used and no adjustment was made for multiple testing. Statistical analyses were performed using SAS (version 9.4; SAS Institute, Cary, NC, USA).

## Results

### Participant Population

**Fig. 1:**
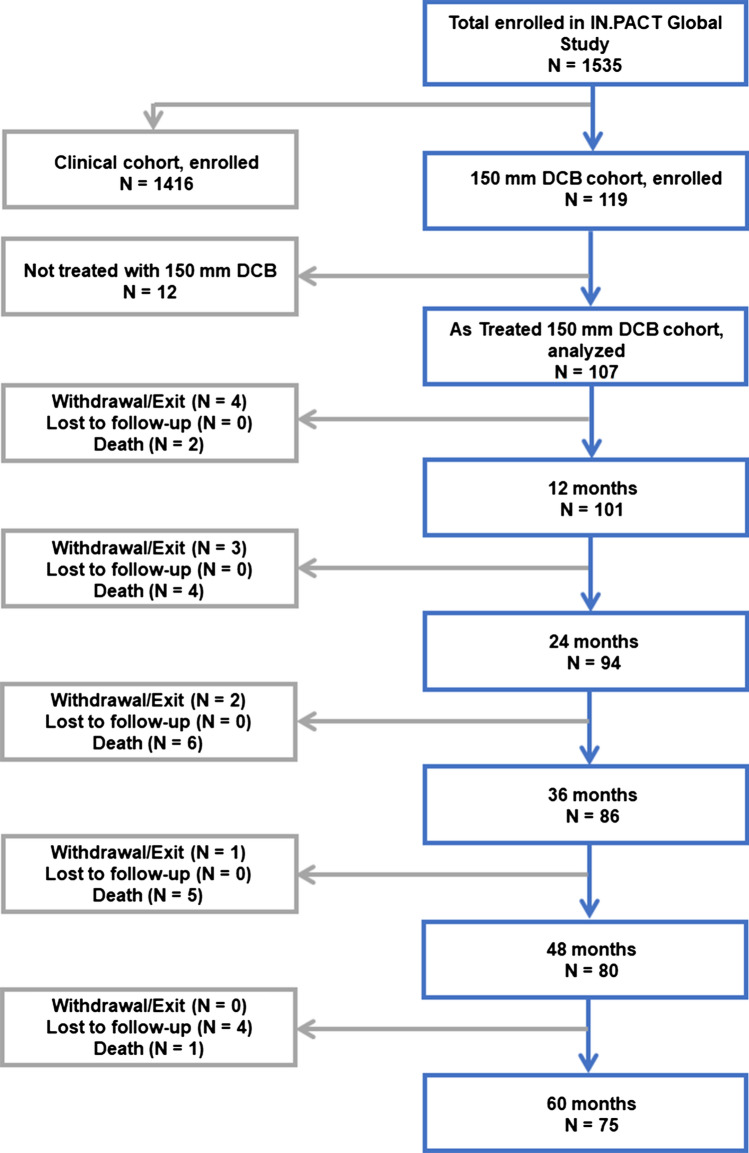
Participant flow in the IN.PACT Global 150 mm DCB cohort through 60 months

In the 150 mm DCB cohort, 119 participants were enrolled, of which 12 patients either did not receive a DCB or did not have all target lesions treated with at least one 150 mm DCB. The remaining 107 participants had all target lesions treated with at least one 150 mm DCB, referred to here as the as-treated 150 mm DCB cohort and included in the analysis (Fig. 1). Baseline demographics and lesion characteristics of the as-treated 150 mm cohort are reported in Table 1 and Table 2. The mean age of participants was 68.0 ± 9.3 years and 76.6% were male. Within this cohort, a significant percentage of participants had diabetes mellitus (41.1%), hyperlipidemia (75.7%), and coronary artery disease (38.8%). The mean lesion length was 20.3 ± 9.2 cm (median 18.0 cm, range 3.0–42.0 cm). Among lesions, 18.0% were in-stent restenosis (ISR) and 58.6% were totally occluded. Most of the lesions were calcified (87.4%) including 17.1% that were severely calcified (circumference ≥ 180° on both sides of the vessel at the same location and lengths greater than or equal to half of the total lesion length). The participant flow through 60 months is shown in Fig. 1. The follow-up compliance rates at 36 and 60 months were 93.0% (80/86) and 92.4% (73/79), respectively.

**Table 1:** Baseline demographic and clinical characteristics in the 107 participants of the IN.PACT global as-treated 150 mm DCB cohort^a,b,c^

Characteristics	IN.PACT Admiral DCB
Age (years)	68.0 ± 9.3 (106)
BMI (kg/m^2^)	28.0 ± 5.6 (105)
Obesity (BMI ≥ 30 kg/m^2^)	27.6% (29/105)
Male	76.6% (82/107)
Hypertension	78.3% (83/106)
Hyperlipidemia	75.7% (78/103)
Diabetes mellitus	41.1% (44/107)
Insulin dependent diabetes mellitus	20.6% (22/107)
Carotid artery disease	24.4% (22/90)
Coronary artery disease	38.8% (38/98)
Current smoker	39.6% (42/106)
Renal insufficiency (baseline serum creatinine ≥ 1.5 mg/dl)	10.3% (9/87)
On dialysis	1.9% (2/107)
Below-the-knee vascular disease of target leg (stenotic/occluded)	47.5% (48/101)
Previous peripheral revascularization	43.9% (47/107)
Iliac	14.0% (15/107)
Common femoral	8.4% (9/107)
Femoral profunda	5.6% (6/107)
Superficial femoral	36.4% (39/107)
Popliteal	8.4% (9/107)
Below the knee	3.7% (4/107)
Previous limb amputation	0.9% (1/107)^d^
Rutherford category	
0	0.0% (0/107)
1	0.0% (0/107)
2	24.3% (26/107)
3	64.5% (69/107)
4	8.4% (9/107)
5	1.9% (2/107)^e^
6	0.9% (1/107)^e^
ABI^f^ (mmHg ratio)	0.653 ± 0.213 (98 limbs)

ABI, ankle-brachial index; BMI, body mass index; DCB, drug-coated balloon

^a^Continuous data are presented as the mean ± standard deviation with the number with data; categorical data are given as the percentage (number/number with data)

^b^Summaries are based on non-missing assessments

^c^Site reported data

^d^There was only one participant with previous limb amputation. This amputation was above the knee

^e^Two participants classified as Rutherford Category 5 and one participant classified as Rutherford Category 6 were enrolled and included in this analysis due to protocol violation

^f^ABI for all target limbs treated are included (can be bilateral)

**Table 2:** Lesion characteristics in the 107 participants of the IN.PACT global as-treated 150 mm DCB cohort^a,b,c,d^

Lesion characteristics	IN.PACT Admiral DCB (*N* = 107 Participants *N* = 111 Lesions)
Pre-procedure	
Lesion type	
De novo	78.4% (87/111)
Restenotic (non-stented)	3.6% (4/111)
In-stent restenosis	18.0% (20/111)
Vessel^e^	
SFA	96.4% (107/111)
PA	31.5% (35/111)
Calcification	
None	12.6% (14/111)
Mild	34.2% (38/111)
Moderate	22.5% (25/111)
Moderately severe	13.5% (15/111)
Severe^f^	17.1% (19/111)
Reference vessel diameter (mm)	5.2 ± 0.5 (111)
Occluded lesion (100% stenosis)	58.6% (65/111)
Diameter stenosis (%)	93.5 ± 10.0 (111)
Lesion length (cm)	20.3 ± 9.2 (111)
Procedure	
Provisional stent rate per participant	36.4% (39/107)
Provisional stent rate per lesion	36.0% (40/111)
Stent coverage in target lesion	
Spot stenting	20.0% (8/40)
Partial lesion coverage	47.5% (19/40)
Whole lesion coverage	32.5% (13/40)
Reason for provisional stenting	
Persistent residual stenosis ≥ 50%	60.0% (24/40)
Flow-Limiting dissection^g^	45.0% (18/40)
Other	10.0% (4/40)
Pre-dilatation	88.8% (95/107)
Post-dilatation	47.7% (51/107)
Post-procedure	
Residual stenosis (%)	10.7 ± 12.3 (111)
Dissection grade	
0 (no dissection)	54.1% (60/111)
A	11.7% (13/111)
B	13.5% (15/111)
C	9.9% (11/111)
D	6.3% (7/111)
E	3.6% (4/111)
F	0.9% (1/111)

DCB, drug-coated balloon; PA, popliteal artery; SFA, superficial femoral artery

^a^Continuous data are presented as the mean ± standard deviation with number with data; categorical data are given as the percentage (number/number with data)

^b^Summaries are based on non-missing assessments

^c^Site reported data

^d^The IN.PACT Global Study allowed bilateral (two target limbs) treatment and multiple target lesions in each target limb to be treated

^e^Multiple lesion locations are reported in a single target limb, the total lesion locations could be more than the total number of target limbs

^f^Defined as circumference ≥ 180° on both sides of the vessel at the same location and lengths greater than or equal to half of the total lesion length [15]

^g^Dissection classification was based on the National Heart, Lung, and Blood Institute classification [48]

### Effectiveness and Safety Outcomes Through 36 Months

**Fig. 2:**
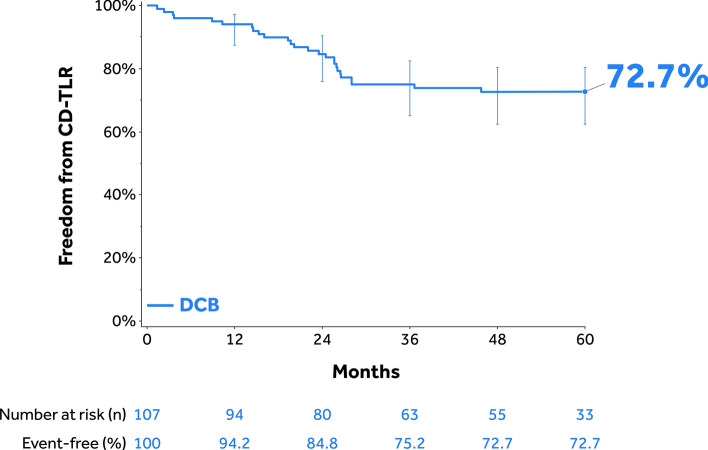
Kaplan–Meier estimate of freedom from CD-TLR through 60 months in the IN.PACT Global as-treated 150 mm DCB cohort. Bars represent the 95% confidence intervals. CD-TLR, clinically driven target lesion revascularization. DCB, drug-coated balloon

**Fig. 3:**
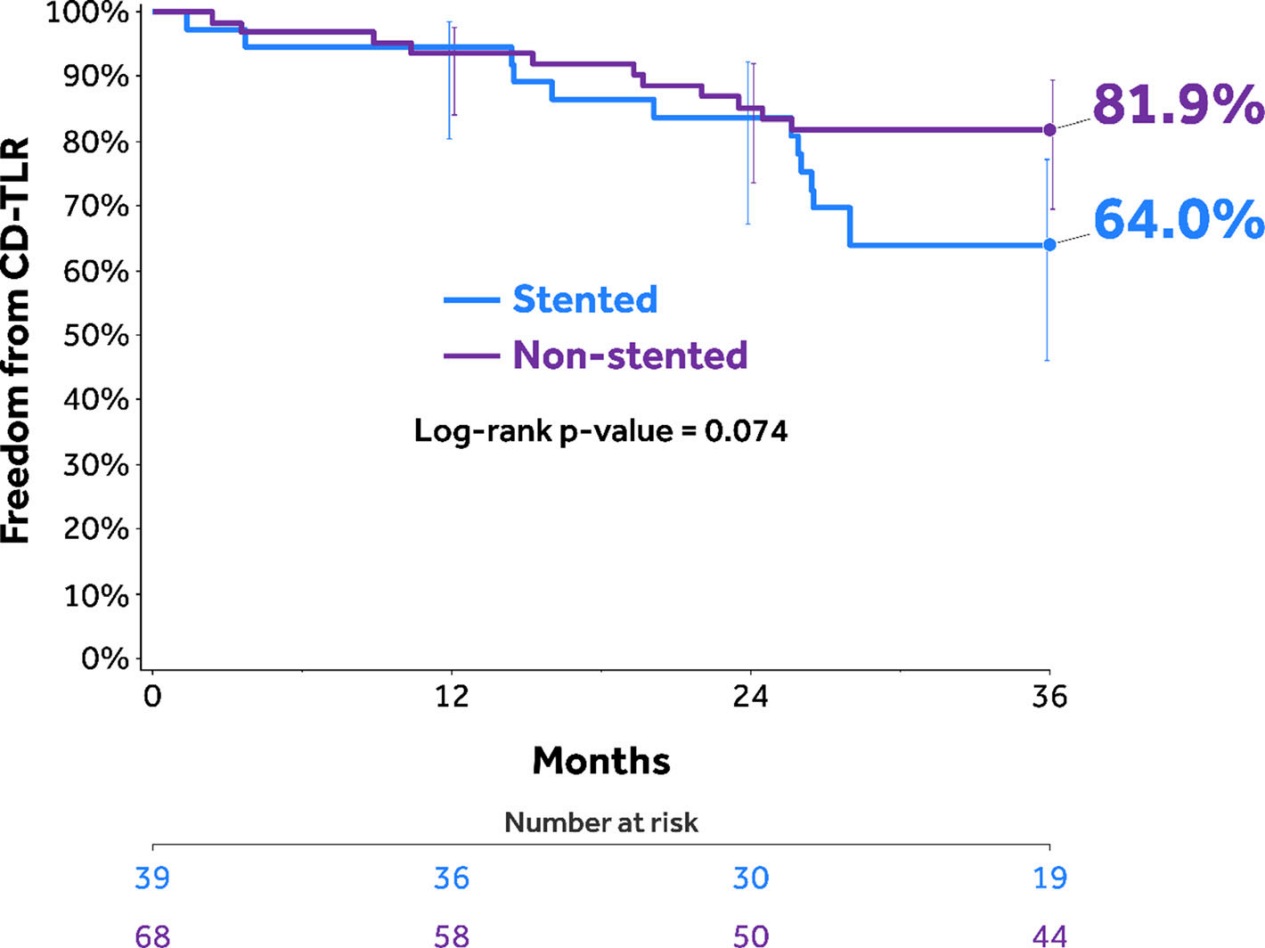
Kaplan–Meier estimate of freedom of CD-TLR through 36 months in the stented versus non-stented subsets of the IN.PACT Global as-treated 150 mm DCB cohort. The baseline characteristics of these two subsets are reported in Supplementary Table 1. Bars represent the 95% confidence intervals. CD-TLR, clinically driven target lesion revascularization

The Kaplan–Meier estimate of freedom from CD-TLR in the 150 mm DCB cohort was 75.2% (95% CI: 65.2%–82.6%; Fig. 2). The RMST to first CD-TLR with a time horizon of 36 months was 948.4 days (95% CI: 897.0–999.8 days; Table 3). Primary and secondary sustained clinical improvement rates were 65.5% (55/84) and 82.7% (67/81), respectively (Table 3). The Kaplan–Meier estimate of freedom from CD-TLR in the stented versus non-stented subgroups within the as-treated 150 mm cohort was 64.0% (95% CI 46.1%–77.3%) versus 81.9% (95% CI 69.7%–89.6%; log-rank *p* = 0.074; Fig. 3), respectively.

**Table 3:** Effectiveness and safety outcomes through 36 months^a^

	IN.PACT Global as-treated 150 mm DCB Cohort (N = 107 Participants)
Effectiveness parameters	
Primary sustained clinical improvement^b^	65.5% (55/84)
Secondary sustained clinical improvement^c^	82.7% (67/81)
Immediate hemodynamic improvement at post-procedure^d^	87.1% (81/93)
Sustained hemodynamic improvement^e^	56.3% (40/71)
Device success^f^	99.0% (205/207)
Procedural success^g^	100.0% (111/111)
Clinical success^h^	99.1% (106/107)
Safety parameters	
Safety composite endpoint (freedom from)	74.2%
Device- and procedure-related death through 30 days^**i**^	0.0% (0)
Target limb major amputation within 36 months^**i**^	1.0% (1)
CD-TVR^j^ within 36 months^**i**^	25.8% (25)
Cumulative complications within 36 months^i^	
MAE composite (death, major target limb amputation, CD-TVR^j^, thrombosis at the target lesion site)	35.8% (36)
Death (all-cause)	13.1% (13)
CD-TVR^j^	25.8% (25)
Major target limb amputation	1.0% (1)
Thrombosis at the target lesion site	2.0% (2)
CD-TLR^k^	24.8% (24)
Any TVR	26.5% (26)
Any TLR	25.6% (25)
Other major secondary 36-month outcome measures	
Restricted mean survival time to first CD-TLR^k^ through 1080 days post-index procedure (days)	948.4 ± 26.2^l^
Change in quality of life from baseline by EQ-5D index	0.220 ± 0.291 (69)
Walking impairment by WIQ (%)	80.2 ± 25.5 (63)
Nights in hospital due to index lesion, days	2.1 ± 3.3 (107)

ABI, ankle-brachial index; CD, clinically driven; DCB, drug-coated balloon; EQ-5D, EuroQol in 5 dimensions; TLR, target lesion revascularization; TVR, target vessel revascularization; WIQ, Walking Impairment Questionnaire

^a^Continuous data are presented as the mean ± standard deviation with number with data; categorical data are given as the percentage (number/number with data). Adverse events were adjudicated by the independent Clinical Events Committee. Unless otherwise specified summaries are based on non-missing assessments

^b^Primary sustained clinical improvement is defined as sustained upward shift of at least 1 category on Rutherford classification as compared to baseline without the need for repeated TLR or surgical revascularization in amputation-free surviving participants

^c^Secondary sustained clinical improvement is defined as sustained upward shift of at least 1 category on Rutherford classification as compared to baseline including the need for repeated TLR or surgical revascularization in amputation-free surviving participants

^d^Immediate hemodynamic improvement is defined as an ABI improvement of ≥ 0.1 or to an ABI ≥ 0.9

^e^Sustained hemodynamic improvement is defined as persistent improvement of ABI values with ≥ 0.1 as compared to baseline values or to an ABI ≥ 0.9 throughout follow-up without the need for repeated TLR or surgical revascularization in amputation-free surviving participants

^f^Device success was defined as successful delivery, inflation, deflation, and retrieval of the intact study balloon device without burst below the rated burst pressure

^g^Procedural success defined as residual stenosis of ≤ 50% (non-stented participants) or ≤ 30% (stented participants) by visual estimate

^h^Clinical success defined as procedural success without procedural complications (death, major target limb amputation, thrombosis of the target lesion, or TVR) prior to discharge

^i^Percentages are cumulative incidence based on Kaplan–Meier estimate (number of patients with events)

^j^CD-TVR is defined as any reintervention at the target vessel due to symptoms or drop of ABI ≥ 20% or > 0.15 when compared with post-index procedure baseline ABI

^k^CD-TLR is defined as any reintervention within the target lesion due to symptoms or drop in ABI ≥ 20% or > 0.15 when compared to post-index procedure baseline ABI

^l^Mean ± Standard error

The safety endpoint, a composite of freedom from device- and procedure-related mortality through 30 days and freedom from major target limb amputation and CD-TVR within 36 months, was 74.2% (Table 3). The cumulative incidence of all-cause death was 13.1% through 36 months. There was only one case of target limb major amputation through 36 months (cumulative incidence rate of 1.0%); this patient was classified as RC 6 at baseline, enrolled due to protocol deviation. The rate of thrombosis at the target lesion site was 2.0%. The cumulative incidence for the MAE composite (all-cause death, major target limb amputation, CD-TVR, thrombosis at the target lesion site) rate was 35.8% through 3 years (Table 3).

### Effectiveness and Safety Outcomes Through 60 Months

**Fig. 4:**
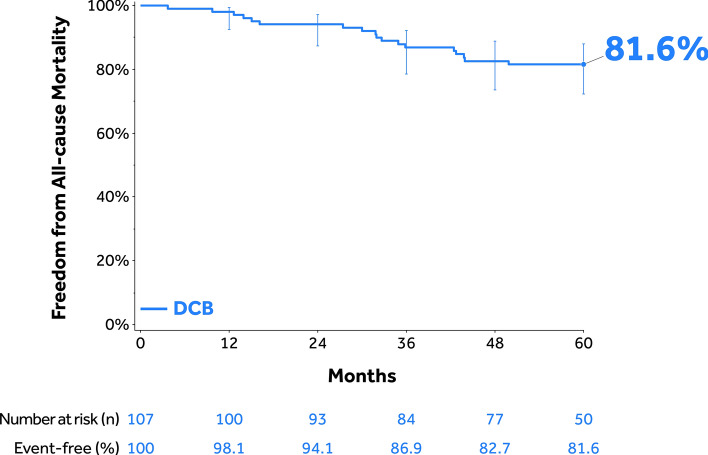
Kaplan–Meier estimate of freedom from all-cause mortality through 60 months in the IN.PACT Global as-treated 150 mm DCB cohort. Bars represent the 95% confidence intervals. DCB, drug-coated balloon

Through 60 months, the Kaplan–Meier estimate of freedom from CD-TLR in the 150 mm DCB cohort was 72.7% (95% CI: 62.4%–80.5%; Fig. 2). The Kaplan–Meier estimate of the safety composite endpoint was 70.5% (Table 4) and the freedom from all-cause mortality was 81.6% (95% CI: 72.4%–88.0%; Fig. 4). There was no additional major target limb amputation between 36- and 60-month follow-up. The cumulative incidence of CD-TVR was 29.5% through 60 months.

**Table 4:** Effectiveness and safety outcomes through 60 months^a^

Parameters	IN.PACT Global as-treated 150 mm DCB Cohort (N = 107 Participants)
Safety parameters	
Safety composite endpoint (freedom from)	70.5%
Device- and procedure-related death through 30 days^b^	0.0% (0)
Target limb major amputation within 60 months^b^	1.0% (1)
CD-TVR^b,c^ within 60 months	29.5% (28)
Cumulative complications within 60 months ^b^	
MAE composite (death, major target limb amputation, CD-TVR^c^, thrombosis at the target lesion site)	44.3% (44)
Death (all-cause)	18.4% (18)
CD-TVR^c^	29.5% (28)
Major target limb amputation	1.0% (1)
Thrombosis at the target lesion site	3.2% (3)
CD-TLR^d^	27.3% (26)
Any TVR	30.2% (29)
Any TLR	28.0% (27)

ABI, ankle-brachial index; CD, clinically driven; DCB, drug-coated balloon; TLR, target lesion revascularization; TVR, target vessel revascularization

^a^Adverse events were adjudicated by the independent Clinical Events Committee

^b^Percentages are cumulative incidence based on Kaplan–Meier estimate (number of patients with events)

^c^CD-TVR is defined as any reintervention at the target vessel due to symptoms or drop of ABI ≥ 20% or > 0.15 when compared with post-index procedure baseline ABI

^d^CD-TLR is defined as any reintervention within the target lesion due to symptoms or drop in ABI ≥ 20% or > 0.15 when compared to post-index procedure baseline ABI

## Discussion

Long lesions, along with chronic total occlusions (CTO) and calcified lesions of the femoropopliteal vessel bed, are seen frequently in daily practice yet are some of the most challenging lesions to treat. PTA performs poorly in longer lesions (> 10 cm) with 1 year restenosis rates of over 70% [16]. High rates of restenosis have also been reported after stenting with bare metal stents (BMS) in long SFA lesions. ISR occurs with a frequency of 20%–40% at 1 year for lesions < 15 cm [17–19]. There is a paucity of data for longer femoropopliteal lesions (> 15 cm), but the rate of ISR in these long lesions after stenting is likely to be upwards of 50%. Furthermore, these restenotic lesions often present with diffuse ISR, which represents an additional challenge.

The current analysis reports outcomes from the pre-specified 150 mm DCB cohort of the IN.PACT Global Study, which is distinct from the IN.PACT Global long lesion imaging cohort (clinical cohort) published previously [8]. In the as-treated 150 mm cohort, participants had all target lesions treated with at least one 150 mm DCB, whereas the balloon size was not restricted in the clinical cohort. Similar to the clinical cohort, participants had complex, calcified, and long femoropopliteal lesions. The results demonstrated sustained safety and clinical benefit of the 150 mm long IN.PACT Admiral DCB for long lesions with low rates of reinterventions and low major amputations through 5 years. Previously, a meta-analysis reported an association between the use of paclitaxel-coated devices and late mortality [20], although patient-level meta-analyses and large real-world registries could not authenticate the findings [21–31]. Nonetheless, to address the concern of long-term safety in this cohort, safety outcomes were analyzed out to 5 years and results showed sustained safety throughout the 5 year follow-up.

There are limited global registries that reported outcomes through 3 or 5 years. We compared the results of the present study to that of RCTs even though the inclusion criteria of RCTs are mostly restricted to less complex and shorter lesions. The Kaplan–Meier estimate of freedom from CD-TLR was 75.2% through 3 years in this 150 mm DCB cohort compared to 84.5% for DCBs in the IN.PACT SFA RCT [5], 83.6%–85.3% for drug-eluting stents (DES) [32; 33] and 69.7%–75.5% for BMS [34; 35]. A 4 year freedom from CD-TLR of 76.7% was reported for the DCB arm in the ILLUMENATE Pivotal study [36]. There were only two reinterventions reported between 3 and 4 years, and none past 4 years; as a result, the freedom from CD-TLR was well sustained through 5 years (72.7%) in this 150 mm DCB cohort. The 5 year freedom from CD-TLR rate (72.7%) was comparable to the freedom from CD-TLR rates of the DCB arm in prior RCTs: 79% in the THUNDER trial [37], 74.5% in the IN.PACT SFA trial [38] and 83.1% in the Zilver PTX RCT [33]. Of note, the mean lesion lengths were much shorter in the aforementioned RCTs (6.6–8.9 cm) compared to the 150 mm cohort of the current study (20.3 cm). The 5 year freedom from CD-TLR of 72.7% in the 150 mm cohort is also favorable compared to the full clinical cohort of the IN.PACT Global Study (69.4%; mean lesion length 12.1 cm) [39], suggesting that treating long lesions with long balloons may be more effective than multiple overlapped balloons.

The provisional stenting rate of 36.4% in this 150 mm cohort is higher than those reported for DCB RCTs [3; 7], which is expected considering the longer mean lesion length (20.3 cm), and the presence of CTO (58.6%) and severely calcified lesions (17.1%) in the 150 mm cohort. Furthermore, this provisional stenting rate is in line with other long lesion cohorts of DCB studies; 35.7% in the long lesion cohort of the Lutonix Global SFA registry [40] and 39.4% in the long lesion imaging cohort of the IN.PACT Global study [41]. The Kaplan–Meier freedom from CD-TLR estimates were numerically higher in the non-stented group (81.9% non-stented vs. 64.0% stented), although the difference was not statistically significant. While the sample sizes were too small to allow any definitive conclusion, it is interesting to see a marked drop in the freedom from CD-TLR at 24 months in the stented subgroup. This may suggest a signal toward decreased effectiveness for provisional stents in long lesions. However, these exploratory findings will need to be confirmed in a larger prospective analysis. The results also suggest that vessel preparation strategies that reduce the need for provisional stenting may improve DCB outcomes further.

Despite the long and complex lesions, the results of this study demonstrated durable safety of treatment with long DCBs in this patient population. The freedom from the safety composite endpoint was high (70.5%) through 5 years. There was only one major target limb amputation (RC 6 at baseline and to be considered a protocol deviation) through 5 years and no device- or procedure-related deaths were found. The cumulative incidence of thrombosis at the target lesion site was also low through 5 years (3.2%). The 5 year all-cause mortality rate of 18.4% in the present study was consistent with the 5 year rates reported (10%–52%) among patients diagnosed with PAD in epidemiologic studies [42; 43]. The 5 year freedom from mortality (survival) rate reported in the present study (81.6%) was also aligned with the rates observed in PAD endovascular studies. Miura et al. reported an 83.4% 5 year survival in an overall claudicant patient population but a survival rate of 53.5% in high-risk claudicant patients. [44] In another retrospective analysis of consecutive patients who underwent femoropopliteal endovascular interventions, Kumins et al. reported 4 year survival rates of 80.7%–46.6% in patients treated with paclitaxel-coated devices and survival rates of 64.4%–32.8% in patients treated with non-paclitaxel devices [45]. The authors also concluded that the paclitaxel-treated group had an improved survival rate compared to the non-paclitaxel group in younger patients (< 60 years). Previously, two pool analyses reports have demonstrated the lack of an association between mortality and this DCB [46; 47].

Limitations: This is a prospective and pre-specified cohort, yet it was enrolled non-consecutively. Other limitations include the single-arm design of the study and a lack of an active comparator group, unlike RCTs. Therefore, the results could not be compared directly with other modalities. Clinic visits were conducted through 3 years; however, only phone follow-up data were available for 4 and 5 years. Anatomical data were not available for analysis of follow-up outcomes in this cohort.

## Conclusion

The results of this analysis demonstrate sustained long-term safety of the 150 mm IN.PACT Admiral DCB for the treatment of obstructive, long femoropopliteal atherosclerotic lesions in real-world patients. The results also show that DCB angioplasty is an effective revascularization modality in long complex lesions.

## Impact on Daily Practice

There are limited long-term publications on the safety and effectiveness of paclitaxel-coated DCBs for the treatment of long and complex femoropopliteal atherosclerotic lesions in the real-world setting. The long-term outcomes from this global cohort showed that the 150 mm IN.PACT Admiral DCB is safe with no device- or procedure-related deaths, has low rates of amputation, and is highly effective as a standalone therapy or in conjunction with provisional stenting in the treatment of long SFA and popliteal complex lesions.

## Supplementary Information

 Below is the link to the electronic supplementary material.

Supplementary file1 (DOCX 53 kb)

## Abbreviations

ABI: Ankle-Brachial Index
BMS: Bare Metal Stent
CEC: Clinical Events Committee
CD-TLR: Clinically Driven Target Lesion Revascularization
CD-TVR: Clinically Driven Target Vessel Revascularization
CTO: Chronic Total Occlusion
DCB: Drug-Coated Balloon
DES: Drug-Eluting Stent
ISR: In-Stent Restenosis
MAE: Major Adverse Event
PAD: Peripheral Artery Disease
PTA: Percutaneous Transluminal Angioplasty
RC: Rutherford Category
RCT: Randomized Controlled Trial
SFA: Superficial Femoral Artery
