# Supplementary material for: Long-Term Outcomes of the 150 mm Drug-Coated Balloon Cohort from the IN.PACT Global Study
Source: Cardiovasc Intervent Radiol. 2022 Jul 21;45(9):1276–87. doi: 10.1007/s00270-022-03214-y (PMC9458561; doi:10.1007/s00270-022-03214-y)
Supplement: Supplementary file 1 — Supplementary file1 (DOCX 53 kb) [file 270_2022_3214_MOESM1_ESM.docx]

**Supplementary Table 1. Baseline Participant and Lesion Characteristics in the Provisional Stented and Non-Stented Participants of the IN.PACT Global As-Treated 150 mm DCB Cohort^a,b,c^**

| **Characteristics** | **IN.PACT DCB Stented (N=39 Subjects) (N=42 Lesions)** | **IN.PACT DCB** **Non-Stented** **(N=68 Subjects)** **(N=69 Lesions)** | **p-value** |
| --- | --- | --- | --- |
| Age (years) | 67.4 ± 8.9 | 68.3 ± 9.5 | 0.630 |
| Obesity (BMI ≥ 30 kg/m^2^) | 20.5% (8/39) | 31.8% (21/66) | 0.262 |
| Male | 74.4% (29/39) | 77.9% (53/68) | 0.813 |
| Hypertension | 73.7% (28/38) | 80.9% (55/68) | 0.463 |
| Hyperlipidemia | 73.0% (27/37) | 77.3% (51/66) | 0.639 |
| Diabetes Mellitus | 35.9% (14/39) | 44.1% (30/68) | 0.423 |
| Carotid Artery Disease | 12.9% (4/31) | 30.5% (18/59) | 0.076 |
| Coronary Artery Disease | 34.3% (12/35) | 41.3% (26/63) | 0.525 |
| Current Smoker | 48.7% (19/39) | 34.3% (23/67) | 0.156 |
| Renal Insufficiency^d^ | 7.1% (2/28) | 11.9% (7/59) | 0.712 |
| Below-the-knee Vascular Disease of Target Leg (Stenotic/Occluded) | 44.4% (16/36) | 49.2% (32/65) | 0.682 |
| Previous Peripheral Revascularization | 46.2% (18/39) | 42.6% (29/68) | 0.840 |
| Previous Limb Amputation | 0.0% (0/39) | 1.5% (1/68) | 1.000 |
| Rutherford Category |  |  | 0.456 |
| 0 | 0.0% (0/39) | 0.0% (0/68) |  |
| 1 | 0.0% (0/39) | 0.0% (0/68) |  |
| 2 | 23.1% (9/39) | 25.0% (17/68) |  |
| 3 | 61.5% (24/39) | 66.2% (45/68) |  |
| 4 | 7.7% (3/39) | 8.8% (6/68) |  |
| 5 | 5.1% (2/39)^e^ | 0.0% (0/68) |  |
| 6 | 2.6% (1/39)^e^ | 0.0% (0/68) |  |
| ABI^f^ (mmHg ratio), per target limb | 0.591 ± 0.148 (37 limbs) | 0.691 ± 0.237 (61 limbs) | 0.011 |
| Lesion Type |  |  | 0.929 |
| De novo | 78.6% (33/42) | 78.3% (54/69) |  |
| Restenotic (non-stented) | 4.8% (2/42) | 2.9% (2/69) |  |
| In-stent Restenosis | 16.7% (7/42) | 18.8% (13/69) |  |
| Vessel^g^ |  |  |  |
| SFA | 97.6% (41/42) | 95.7% (66/69) | 1.000 |
| PPA | 23.8% (10/42) | 36.2% (25/69) | 0.209 |
| Calcification |  |  | 0.831 |
| None | 11.9% (5/42) | 13.0% (9/69) |  |
| Mild | 35.7% (15/42) | 33.3% (23/69) |  |
| Moderate | 19.0% (8/42) | 24.6% (17/69) |  |
| Moderately Severe | 14.3% (6/42) | 13.0% (9/69) |  |
| Severe | 19.0% (8/42) | 15.9% (11/69) |  |
| RVD (mm) | 5.2 ± 0.58 (42) | 5.2 ± 0.53 (69) | 0.765 |
| Occluded Lesion (100% stenosis) | 78.6% (33/42) | 46.4% (32/69) | 0.001 |
| Lesion Length (cm) | 20.3 ± 8.8 (42) | 20.2 ± 9.5 (69) | 0.961 |
| Pre-dilatation | 94.9% (37/39) | 85.3% (58/68) | 0.204 |
| Post-dilatation | 74.4% (29/39) | 32.4% (22/68) | < 0.001 |

Abbreviations: ABI, ankle-brachial index; BMI, body mass index; DCB, drug-coated balloon.

^a^Continuous data are presented as the mean ± standard deviation with the number with data; categorical data are given as the percentage (number/ number with data).

^b^Summaries are based on non-missing assessments.

^c^Site reported data.

^d^baseline serum creatinine ≥ 1.5 mg/dl

^e^Two participants classified as Rutherford Category 5 and one participant classified as Rutherford Category 6 were enrolled and included in this analysis due to protocol violation

^f^ABI for all target limbs treated are included (can be bilateral).
^g^Multiple lesion locations are reported in a single target limb, the total lesion locations could be more than the total number of target limbs.
